# Gene Mapping and Molecular Marker Development for Controlling Purple-Leaf Trait in Pakchoi (*Brassica rapa* subsp. *chinensis* (L.) Hanelt)

**DOI:** 10.3390/genes16101184

**Published:** 2025-10-12

**Authors:** Bo Song, Qinyu Yang, Wenqi Zhang, Xiao Yang, Li Zhang, Lin Ouyang, Limei He, Longzheng Chen, Zange Jing, Tao Huang, Hai Xu, Yuejian Li, Qichang Yang

**Affiliations:** 1 Institute of Urban Agriculture, Chinese Academy of Agricultural Sciences, Chengdu 610213, China; 2 Jiangsu Key Laboratory for Horticultural Crop Genetic Improvement, Institute of Vegetable Crops, Jiangsu Academy of Agricultural Sciences, Nanjing 210014, China; 3 College of Agriculture and Life Science, Kunming University, Kunming 650214, China

**Keywords:** anthocyanin, BSA-Seq, gene mapping, PARMS, marker-assisted selection

## Abstract

Backgrounds: purple pakchoi (*Brassica rapa* subsp. *chinensis* (L.) Hanelt) is rich in anthocyanins, which contribute to its significant edible, ornamental, and potential health-promoting value. Fine mapping of the genes responsible for the purple-leaf trait is essential for establishing molecular marker-assisted breeding and facilitating genetic improvement. Methods: In this study, we used the inbred purple-leaf line ‘PQC’ and green-leaf line ‘HYYTC’ as parents to construct a six-generation genetic segregation population. We analyzed the inheritance pattern of the purple-leaf trait and combined Bulked Segregant Analysis Sequencing (BSA-Seq) with penta-primer amplification refractory mutation system (PARMS) to map the causal gene. Results: the main findings are as follows: the purple-leaf trait is controlled by a single dominant gene. Using BSA-Seq and PARMS, the genes were mapped to a 470 kb region (31.18–31.65 Mb) on chromosome A03. Within this interval, 29 candidate genes were identified, *Bra017888* which encoding trehalose phosphate synthase 10 (TPS10), was highlighted as a potential regulator of anthocyanin biosynthesis. A developed molecular marker, SNP31304070, based on the final candidate region, successfully distinguished between purple homozygous and purple heterozygous plants in the F_2_ and F_3_ populations. Conclusions: the candidate gene controlling purple-leaf trait was finally located to A03 chromosome 31.18–31.65 Mb. The SNP31304070 marker and trait were co-separated, This marker could be applied to molecular-assisted breeding in purple pakchoi.

## 1. Introduction

Pakchoi is an important leafy vegetable in the cruciferous family, and purple pakchoi is rich in anthocyanins and has an ornamental function and is edible for health care. The purple-leaf trait, which directly influences its nutritional and commercial value, has long been a key objective in genetic research and breeding improvement [1,2]. Previous physiological studies have revealed that the purple coloration in leaves arises from variations in anthocyanin composition and distribution, ultimately leading to differences in leaf color [3]. In terms of anthocyanin biosynthesis, transcriptome sequencing analysis of purple pakchoi revealed that eighteen structural genes involved in anthocyanin synthesis and five transcription factor genes were differentially expressed between the purple cultivar ‘Zi Zuan’ and the green cultivar ‘Jing Guan’ [4]. Similarly, Yang et al. (2024) identified twenty structural genes related to anthocyanin biosynthesis and twenty-eight transcriptional regulatory genes that were differentially expressed in purple and green materials [5]. However, due to the large number of genes involved, the key gene primarily responsible for controlling the purple trait in pakchoi remains to be further elucidated.

Utilizing map-based cloning for purple-leaf trait is an effective approach to pinpoint target genes. In Chinese cabbage (*Brassica rapa* subsp. *pekinensis* (Lour.) Hanelt), the key locus controlling the purple-leaf trait was located on the A02 and A09 linkage group [6,7]. Wu et al. (2017) mapped the gene controlling purple leaves to a 47.91 kb interval on chromosome A07 [8]. Further research by He et al. (2016) revealed that *BrMYB2* is located within this interval [9]. In the case of pakchoi, Liu et al. (2023) employed traditional Bulked Segregant Analysis on an F_2_ population derived from a cross between green Chinese cabbage and purple pakchoi, mapping the target gene to the terminal region of linkage group A03, the selection accuracy of two developed molecular markers, BVRCP10-6 and BrID10399, reached 100% [10]. On this basis, Wang et al. (2014) constructed a BC_1_ population and used BSA method to locate the candidate genes between InDel markers BVRCPI613 and BVRCPI431, with the location interval being 30.77–30.82 Mb on chromosome A03, and *Bra017831* (*BrLBD39*) presumed as a candidate gene [11]. Guo (2014) constructed an F_2_ population by hybridizing purple pakchoi with flowering Chinese cabbage (*Brassica rapa* var. *parachinensis*), and used BSA-Seq method to map candidate genes to the end of chromosome A03 between 29.68 and 31.18 Mb, including anthocyanin biosynthesis gene *BrCHI3* and regulatory gene *BrLBD39* [12]. The above results showed that the gene controlling purple leaves of pakchoi was located at the end of chromosome A03; however, it remains undetermined which specific gene governs the formation of the purple-leaf trait in pakchoi. Additionally, only a limited number of conventional InDel and SSR molecular markers have been developed, resulting in relatively low identification efficiency.

SNPs (Single Nucleotide Polymorphisms) offer key advantages over InDels and SSRs due to their superior abundance and stability in genomes. Their biallelic nature, while less polymorphic per locus than multi-allelic SSRs, enables simpler, higher throughput, automated scoring on array or sequencing platforms [13,14]. To address these issues, SNP markers were subsequently developed to further narrow the interval. Final candidate gene identification through gene annotation, and SNP markers in the localization interval were used to assist the selection of hybrid progeny. The research establishes a good foundation for gene function analysis and molecular marker-assisted breeding.

## 2. Materials and Methods

### 2.1. Experimental Material

Purple inbred line ‘PQC’ (P_1_) and green inbred line ‘HYYTC’ (P_2_) originated from Jiangsu Academy of Agricultural Sciences. The two lines were cultivated in an insect-proof net house at the Luhe Base, Vegetable Research Institute, Jiangsu Academy of Agricultural Sciences. They were sown in September 2018, transplanted in October, and maintained under moist soil conditions. Cross-pollination was performed at flowering stage in March to generate the F_1_ population. In spring 2019, F_1_ was self-pollinated at flowering phase to produce an F_2_ population. Additionally, F_1_ plants were backcrossed with P_1_ and P_2_ to construct BC_1_P_1_ and BC_1_P_2_ populations, respectively. In autumn 2020, all six generations (P_1_, P_2_, F_1_, F_2_, BC_1_P_1_, and BC_1_P_2_) were cultivated. Leaves were collected at the three-leaf stage, and 0.1 g of each were taken, labeled sequentially, and stored at −70 °C for DNA extraction.

### 2.2. Character Statistics

Leaf color (purple or green) was visually assessed at the three-leaf stage. Segregation ratios of purple-to-green plants in the F_2_, BC_1_P_1_, and BC_1_P_2_ populations were analyzed. Chi-square tests were performed using SPSS 20.0 software (https://www.ibm.com/support/pages/spss-statistics-20-available-download, accessed on 17 May 2021) to evaluate the fit of observed ratios to expected Mendelian ratios. Chi-squared tests were performed at a significant level of 0.05 (χ^2^ 0.05 = 3.84).

### 2.3. DNA Extraction

Genomic DNA was extracted using a modified CTAB method [15]. This optimized CTAB method for plant leaves uses 100 mg tissue ground in liquid nitrogen, lysed for 30 min at 60 °C in a buffer containing 2% CTAB, 2% PVP-40, and 1% sodium sulfite (replacing β-mercaptoethanol), followed by two organic extractions—first with phenol–chloroform–isoamyl alcohol (25:24:1) and second with chloroform–isoamyl alcohol (24:1), then DNA is precipitated at −20 °C for 1 h with 0.7 vol cold isopropanol plus 0.1 vol 5 M NaCl, washed twice with 70% ethanol, air-dried, and re-suspended in TE buffer, yielding high-purity genomic DNA suitable for downstream PCR.

### 2.4. BSA-Seq Analysis

We randomly selected thirty purple-leaf and thirty green-leaf plants from 805 F_2_ individuals to construct extreme pools (P-bulk and G-bulk). Sequencing data were processed by removing adapter-contaminated and low-quality paired-end reads. Using GATK3.3 software, clean reads from two pools were compared to the reference genome of Chinese cabbage (https://plants.ensembl.org/Brassica_rapa_ro18/Info/Cultivars, accessed on 18 July 2023). SNPs were developed using SAMtools (v1.17). These SNPs were used to calculate the SNP-index for both pools (G-bulk and P-bulk). The Δ (SNP-index) was derived as Δ (SNP-index) = SNP-index (P-bulk) − SNP-index (G-bulk). SNP-index = 0.5 indicates equal allele contributions from both parents, suggesting the absence of the candidate gene at that locus. Δ (SNP-index) > 0 signifies genomic regions harboring candidate genes [16].

### 2.5. Mapping the Gene of Controlling Purple-Leaf Trait

Based on the preliminary candidate region identified by BSA-Seq, primers were designed for SNP loci within the interval using the penta-primer amplification refractory mutation system (PARMS). The PARMS master mixture was purchased from Wuhan Gentides Biotech Co., Ltd., Wuhan, China. The PCR-based PARMS assay was performed as follows: 10 μL PCR reaction system (2 × PARMS main mixture 5 μL, allele-specific 1 at 10 μM concentration 0.15 μL, allelic primer 2 at 10 μM concentration 0.15 μL, universal primer at 10 μM concentration 0.4 μL, 50 ng DNA template 1 μL and 3.3 μL ddH_2_O); Drop PCR (denaturation: 94 °C for 20 s, annealing: 65 °C to 57 °C (0.8 °C per cycle) for 1 min, 10 cycles; 94 °C for 20 s, 57 °C for 1 min, 30 cycles). Fluorescence signals were detected using a TECAN Infinite M1000 microplate reader and analyzed with the online tool Snpdecoder (http://www.snpway.com/snpdecoder, accessed on 9 September 2021) to generate genotype plots. A: Homozygous for the ‘PQC’ allele. B: Homozygous for the ‘HYYTC’ allele. H: Heterozygous. Polymorphic SNP markers were screened in the parental lines (‘PQC’ and ‘HYYTC’), F_1_ and F_2_ plants. Recombinants in the F_2_ population were identified by combining phenotypic data with genotyping results. Newly developed SNP markers were used to further genotype recombinants (primer sequences are listed in Supplementary Table S1). The candidate region from fine mapping was analyzed using the Chinese cabbage reference genome. Genes within the interval were annotated, and candidate genes were predicted based on functional annotations.

## 3. Results and Analysis

### 3.1. Genetic Inheritance of the Purple-Leaf Trait 

**Figure 1:**
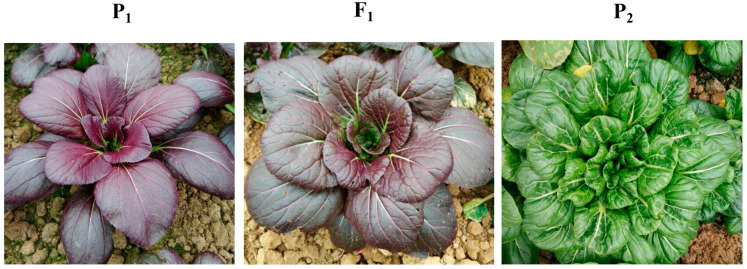
The leaf color of P_1_, P_2_, and F_1_.

Leaf color observations across six generations revealed the following: P_1_ (‘PQC’) exhibited deep purple leaves, P_2_ (‘HYYTC’) displayed green leaves, and F_1_ hybrids showed light purple leaves, intermediate between the parental lines (Figure 1). Segregation analysis in the F_2_ population (5472 plants) identified 3998 purple-leaf and 1474 green-leaf plants, fitting a 3:1 ratio (χ^2^ = 1.81, *p* = 0.577). In the BC_1_P_2_ population (705 plants), 373 purple-leaf and 332 green-leaf plants followed a 1:1 ratio (χ^2^ = 1.61, *p* = 0.232). All individuals in the BC_1_P_1_ population (86 plants) were purple leaf (Table 1). The results showed that the purple-leaf trait was a quality trait controlled by a single dominant gene.

### 3.2. BSA-Seq Analysis Identifies Candidate Region for Purple-Leaf Gene

About 160 G of total reads were obtained in P-bulk, with 95.51% aligned to the reference genome and an average depth of 31×. About 153 G of total reads were obtained in G-Bulk, with 95.48% alignment rate and an average depth of 32× (Table 2).

**Figure 2:**
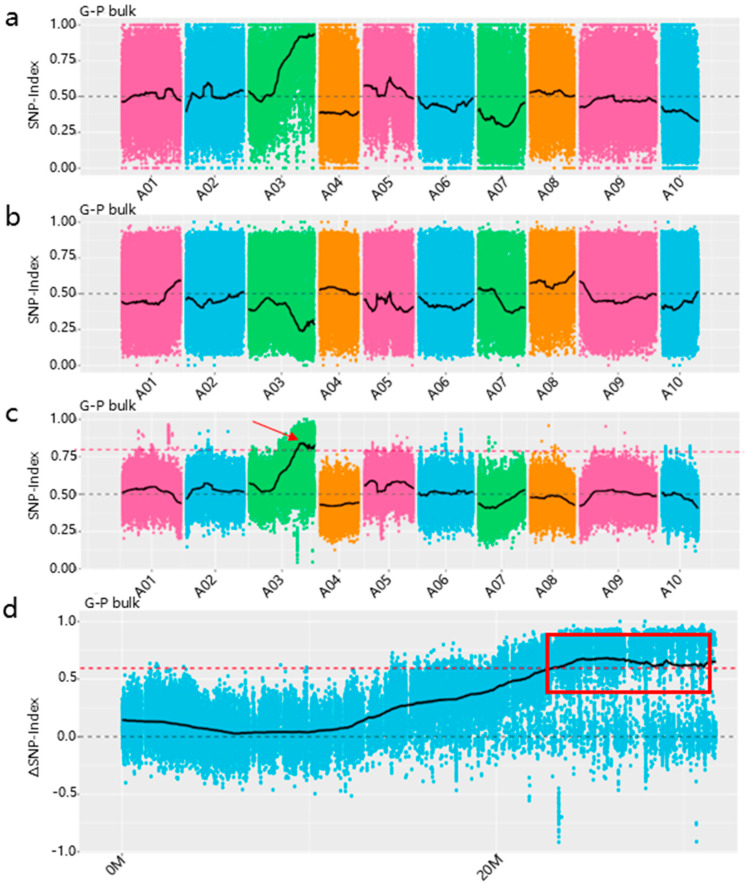
Distribution of ΔSNP−index in chromosomes and identification of genomic region of candidate gene. (**a**) Distribution of SNP-index across all 10 chromosomes in G−bulk sample; (**b**) distribution of SNP−index across all 10 chromosomes in P−bulk sample; (**c**) distribution of Δ (SNP−index) across all 10 chromosomes, with red arrows indicating the mapped interval. (**d**) Distribution of Δ (SNP−index) on chromosome A03. The red dashed line represents the 95 % confidence threshold. The red box highlights the region where Δ (SNP−index) exceeds the threshold, corresponding to the candidate gene interval.

Sequences from both parental lines were aligned to the Chinese cabbage reference genome, identifying 2,776,536 SNPs. Δ (SNP-index) plot across genomic regions (95% confidence level) identified a 7.8 Mb candidate interval (23.88–31.68 Mb) on chromosome A03 (Figure 2).

### 3.3. Fine Mapping of the Purple-Leaf Gene

**Figure 3:**
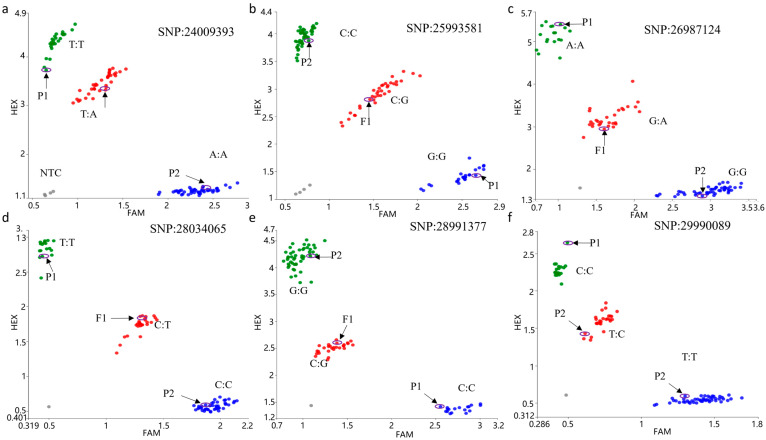
Genotyping of P_1_, P_2_, F_1_, and F_2_ population with six markers. Green dots represent homozygous purple. Red dots represent heterozygous type. Blue dots represent the homozygous green. Grey dots represent undetected sample (NTC). (**a**) Genotyping with SNP24009393 marker; (**b**) Genotyping with SNP25993581 marker; (**c**) Genotyping with SNP26987124 marker; (**d**) Genotyping with SNP28034065 marker; (**e**) Genotyping with SNP28991377 marker; (**f**) Genotyping with SNP29990089marker.

Due to the large size of initial mapping interval, six primer pairs were designed between 24 Mb and 30 Mb (primer sequences in Supplementary Table S1) and amplified in the parental lines, F_1_, and F_2_ plants (44 green and 44 purple individuals). It showed good genotyping results, and the linkage between markers in this interval and the purple trait confirmed the reliability of the BSA-derived candidate region (Figure 3).

To identify recombinant individuals, SNP24009393 and SNP29990089 markers were used to genotype 995 F_2_ plants (700 green-leaf and 295 purple-leaf), yielding 145 recombinants. Notably, eight recombinants (A01-A06, A05-A06, A05-F10, A06-D01, A06-D06, A11-C11, A11-D05, and A11-H06) exhibited green phenotypes but heterozygous genotypes (H) at SNP29990089, indicating that the SNP29990089 marker could not completely distinguish the genotype of the progeny. This suggested the target gene lies downstream of SNP29990089. A terminal marker, SNP31686432, was designed to validate these eight recombinants. All showed homozygous recessive (B) genotypes, confirming the gene location between SNP29990089 and SNP31686432 (Supplementary Table S2).

Twelve additional markers were developed within this interval. Recombinants A01-A06 displayed heterozygous (H) genotype at SNP30820924, narrowing the interval to 30.82–31.68 Mb (Table 3).

**Figure 4:**
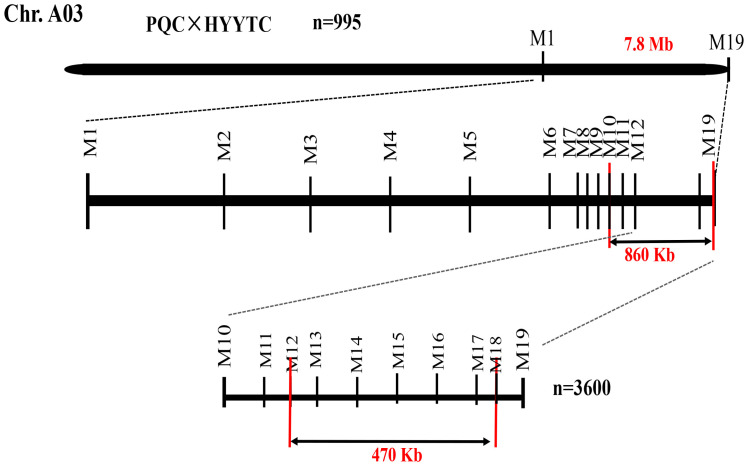
Map-based cloning for candidate genes.

Given the remaining size of the candidate interval, an expanded population of 3600 F_2_ plants (800 green and 2800 purple) was screened using markers SNP30820924 and SNP31686432. Genotyping revealed five recombinants (A12-F09, A32-B04, A34-D12, and A52-H02). Those are purple phenotypes with homozygous recessive (B) genotypes at both markers, while A37-A03 is a purple phenotype but heterozygous (H) at SNP30820924. These five recombinants were further analyzed with additional markers (SNP31209409, SNP31304070, SNP31409624, SNP31521617, and SNP31632032). Genotypes of all five plants perfectly matched their phenotypes, narrowing the final candidate interval to 31.18–31.65 Mb (Table 3, Figure 4).

The 470 kb interval contains 29 genes based on the Chinese cabbage reference genome annotation, including 16 annotated genes and 13 uncharacterized genes (Table 4). Notably, *Bra017888* encodes trehalose phosphate synthase 10 (TPS10). Previous studies have shown that anthocyanin content increased significantly in *Arabidopsis* seedlings treated with trehalose of different concentrations, indicating that trehalose promoted anthocyanin accumulation in plants [17,18], suggesting *Bra017888* may regulate anthocyanin biosynthesis.

### 3.4. Molecular Marker Development

**Figure 5:**
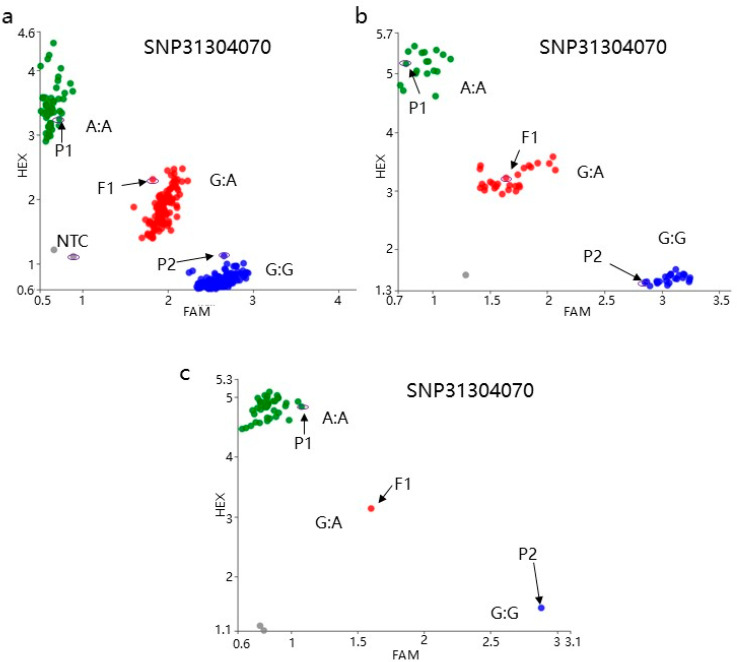
Genotyping of F_2_ and F_3_ population with SNP31304070 marker. (**a**) Genotyping of F_2_ population. (**b**) Genotyping of F_3_-1 population. (**c**) Genotyping of F_3_-2 population. Green dots represent homozygous purple (A). Red dots represent heterozygous type (H). Blue dots represent the homozygous green (B). Grey dots represent undetected sample (NTC).

**Figure 6:**
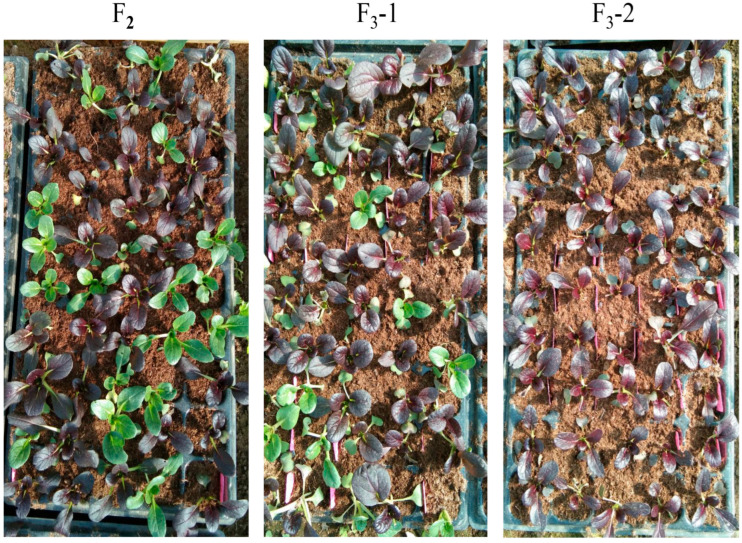
Phenotypic identification of F_2_ and F_3_. F_3_-1: descendants derived from heterozygous purple plant in F_2_; F_3_-2: descendants derived from homozygous purple plant in F_2_.

Within the final mapped interval, five SNP markers (SNP31209409, SNP31304070, SNP31409624, SNP31521617, and SNP31632032) were identified. The representative marker SNP31304070 was selected for validation in the F_2_ and F_3_ populations. Detailed marker information is provided in Supplementary Table S1. Ninety F_2_ plants were randomly selected for genotyping using the SNP31304070 marker. Plants with heterozygous purple (H, red dots) and homozygous purple (A, green dots) genotypes were self-pollinated to generate two F_3_ populations. F_3_-1 was derived from heterozygous purple plants. Among 63 F_3_ individuals, 49 exhibited purple-leaf and 17 showed green-leaf, indicating persistent segregation. F_3_-2 was derived from homozygous purple plants, and all 50 F_3_ plants displayed uniform purple-leaf, confirming the absence of segregation. Genotyping of both F_3_ populations with SNP31304070 revealed 100% concordance between phenotypes and genotypes. This validated the marker’s utility for molecular marker-assisted breeding (Figure 5 and Figure 6).

## 4. Discussion

### 4.1. The Genetic Characteristics of the Gene Controlling Purple-Leaf Trait

Guo (2014) investigated leaf color segregation in an F_2_ population derived from an inter-varietal hybridization between purple pakchoi with flowering Chinese cabbage, the observed ratio of green to purple plants was 1:3, with pure green, intermediate, and pure purple plants segregating at 1:2:1 [12]. This suggested that the purple-leaf trait in pakchoi is controlled by a single dominant gene with incomplete dominance. Similarly, Zhang et al. (2011) reported variations in purple intensity in a genetic population derived from an inter-subspecific hybridization between Chinese cabbage and purple pakchoi, further supporting monogenic dominant inheritance [19]. While these studies utilized inter-subspecific cross or inter-varietal hybridization, in this study, our findings based on intra-varietal hybridization between different inbred lines of pakchoi align with their conclusions, reinforcing the single-gene dominant inheritance model for purple-leaf trait.

### 4.2. Mapping Interval and Candidate Gene Analysis

The above research results indicate that due to the different sources of the purple gene, the target genes controlling anthocyanin synthesis in Chinese cabbage may be located on chromosomes A02, A03, A07, and A09 [6,7,8,9]. In terms of specific gene exploration, sequence analysis indicated that a large insertion in the first intron of the *BrMYB2* gene in green Chinese cabbage suppresses its expression, thereby preventing anthocyanin accumulation [9]. In the case of pakchoi, the gene controlling purple-leaf trait was consistently localized to the terminal region of chromosome A03 [11,12]. Key candidates included *Bra017728* (*BrCHI3*) and *Bra017831* (*BrLBD39*), and sequence analysis revealed a 48 bp deletion in the first exon of *BrCHI3* in purple lines, while *BrLBD39* showed no coding sequence (CDS) differences but exhibited higher expression in green mutants [12,20]. However, our study mapped the candidate interval to a downstream region on A03 (31.18–31.65 Mb), and *BrCHI3* and *BrLBD39* were absent in this interval, and RNA-seq analysis detected no expression of these genes in either parental line, suggesting they are not causal for the purple trait [5]. This discrepancy may stem from differences in genetic materials or population structures across studies. The annotated gene *Bra017888* encodes trehalose phosphate synthase 10 (TPS10). Trehalose, a non-reducing disaccharide, is synthesized via its precursor trehalose-6-phosphate (T6P), catalyzed by TPS enzymes. T6P serves as a carbon availability signal, promoting anthocyanin biosynthesis under high carbohydrate conditions. In Arabidopsis, the TPS gene family comprises 11 members, divided into AtTPS1-4 and AtTPS5-11 subfamilies [21]. While TPS1 has been extensively studied, overexpression in potato and maize increased anthocyanin content by about 2-fold and upregulated MYB/bHLH regulators [22]. Zhao (2018) demonstrated that *JcTPS1* overexpression from *Jatropha* induced anthocyanin-related genes (*AtDFR*, *AtLDOX*) in *Arabidopsis* [23]. Given its positional candidacy and homology to TPS1, *Bra017888* (TPS10) may similarly regulate anthocyanin pathways. Functional validation of this candidate gene should be conducted to further elucidate its role. Additionally, within this mapped interval, certain candidate genes remain unannotated and warrant further attention.

### 4.3. Application of Molecular Marker for Purple-Leaf Trait

During the hybridization and breeding process of purple pakchoi, since the purple color is a dominant trait and green is recessive, heterozygous plants exhibit purple coloration. This makes it difficult to distinguish between homozygous purple and heterozygous purple plants based solely on color. When heterozygous plants are selected during population segregation, their offspring will continue to exhibit color segregation, resulting in a prolonged breeding cycle and reduced selection efficiency. The SNP31304070 marker developed in this study enables precise discrimination of homozygous purple plants and heterozygous purple plants, achieving 100% phenotype–genotype concordance in F_2_ and F_3_ populations.

## 5. Conclusions

In conclusion, the genes controlling purple-leaf trait were finally located to A03 chromosome 31.18-31.65 Mb. *Bra017888* is a candidate gene, indicating a regulatory relationship with the biosynthesis process of anthocyanin.. The SNP31304070 marker and trait were co-separated. This molecular marker will significantly enhance selection efficiency, shorten breeding cycle, and support rapid improvement of anthocyanin-rich varieties in pakchoi.

## Figures and Tables

**Table 1:** Segregation analysis of purple-leaf and green-leaf in the F_2_, BC_1_P_1_, and BC_1_P_2_ population.

Population	Total Plants	Purple-Leaf Plants	Green-Leaf Plants	Expected Ratio	χ^2^	*p* Value
F_2_	5472	3998	1474	3:1	1.81	0.577
BC_1_P_2_	705	373	332	1:1	1.61	0.232
BC_1_P_1_	86	86	0	1:0	-	-

**Table 2:** Sequencing data quality control metrics for P-bulk and G-bulk samples.

Sample	Total Reads (bp)	Mapping Rate (%)	Average Depth (X)	Properly Mapped (%)	Cov_Ratio_10X (%)
P-bulk	159897960	95.48	32	77.74	85.69
G-bulk	153422874	95.51	31	77.68	86.04

**Table 3:** Recombinants and their genotypes were detected in the F_2_ population.

	M1	M2	M3	M4	M5	M6	M7	M8	M9	M10	M11	M12	M13	M14	M15	M16	M17	M18	M19	Color
Mark position	24009393	25993581	26987124	28034065	28990089	29990089	30100772	30402945	30600586	30820924	31150266	31180314	31209409	31304070	31409624	31521617	31632032	31655211	31686432	-
Recombinants	13	13	13	13	13	13	13	5	5	5	5	5	0	0	0	0	0	9	9	-
P_1_	A	A	A	A	A	A	A	A	A	A	A	A	A	A	A	A	A	A	A	purple
P_2_	B	B	B	B	B	B	B	B	B	B	B	B	B	B	B	B	B	B	B	green
F_1_	H	H	H	H	H	H	H	H	H	H	H	H	H	H	H	H	H	H	H	purple
A01-A06	H	H	H	H	H	H	H	H	H	H	B	B	B	B	B	B	B	B	B	green
A05-A06	H	H	H	H	H	H	H	B	B	B	B	B	B	B	B	B	B	B	B	green
A05-F10	H	H	H	H	H	H	H	B	B	B	B	B	B	B	B	B	B	B	B	green
A06-D01	H	H	H	H	H	H	H	B	B	B	B	B	B	B	B	B	B	B	B	green
A06-D06	H	H	H	H	H	H	H	B	B	B	B	B	B	B	B	B	B	B	B	green
A11-C11	H	H	H	H	H	H	H	B	B	B	B	B	B	B	B	B	B	B	B	green
A11-D05	H	H	H	H	H	H	H	B	B	B	B	B	B	B	B	B	B	B	B	green
A11-H06	H	H	H	H	H	H	H	B	B	B	B	B	B	B	B	B	B	B	B	green
A12-F09	B	B	B	B	B	B	B	B	B	B	B	B	H	H	H	H	H	B	B	purple
A32-B04	B	B	B	B	B	B	B	B	B	B	B	B	H	H	H	H	H	B	B	purple
A34-D12	B	B	B	B	B	B	B	B	B	B	B	B	H	H	H	H	H	B	B	purple
A52-H02	B	B	B	B	B	B	B	B	B	B	B	B	H	H	H	H	H	B	B	purple
A37-A03	B	B	B	B	B	B	B	B	B	B	B	B	H	H	H	H	H	H	H	purple

**Table 4:** Gene prediction for candidate interval.

Gene ID	Start	End	Annotation
*Bra017876*	31180442	31180972	Ribosomal protein L23 family protein
*Bra017877*	31181511	31183086	Unknown protein
*Bra017878*	31184432	31185220	Unknown protein
*Bra017879*	31190884	31191666	AP2 domain-containing transcription factor
*Bra017880*	31213843	31214996	Unknown protein
*Bra017881*	31227608	31229937	Pentatricopeptide (PPR) repeat-containing protein
*Bra017882*	31239747	31246015	UDP-glucosyl transferase 75B2
*Bra017883*	31255797	31257231	Agenet domain-containing protein
*Bra017884*	31260954	31266012	Unknown protein
*Bra017885*	31322931	31323275	Unknown protein
*Bra017886*	31341268	31341483	Unknown protein
*Bra017887*	31344356	31346341	Unknown protein
*Bra017888*	31348727	31351464	Trehalose phosphate synthase 10
*Bra017889*	31354005	31356742	Formin homology 2 domain-containing protein
*Bra017890*	31356980	31361049	E1 alpha subunit of the pyruvate dehydrogenase complex
*Bra017891*	31362626	31367740	SIN3-like 5
*Bra017892*	31372380	31372692	5-Aminolevulinic acid dehydrtase 1
*Bra017893*	31383010	31384373	Unknown protein
*Bra017894*	31386945	31387686	Leucine-rich repeat protein kinase
*Bra017895*	31391442	31392231	Unknown protein
*Bra017896*	31417632	31423472	Unknown protein
*Bra017897*	31437219	31450288	Aminophosphlipid ATPase 3
*Bra017898*	31472604	31473209	Thioredoxin H-type 7
*Bra017899*	31495945	31497816	Pentatricopeptide Repeat Protein
*Bra017900*	31522225	31530698	UDP-xylosyltransferase
*Bra017901*	31540532	31547659	Dynamin-like 3
*Bra017902*	31570538	31571898	Unknown protein
*Bra017903*	31590827	31591348	Unknown protein
*Bra017904*	31594274	31595090	Unknown protein

## Data Availability

The original contributions presented in the study are included in the article/Supplementary Materials, further inquiries can be directed to the corresponding authors.

## Supplementary Materials

The following supporting information can be downloaded at: https://www.mdpi.com/article/10.3390/genes16101184/s1; Table S1: PARMS Primer sequence. Table S2: 145 recombinants and their genotypes detected in F_2_ population.
