# Supplementary material for: Gene Mapping and Molecular Marker Development for Controlling Purple-Leaf Trait in Pakchoi (Brassica rapa subsp. chinensis (L.) Hanelt)
Source: Genes (Basel). 2025 Oct 12;16(10):1184. doi: 10.3390/genes16101184 (PMC12563174; doi:10.3390/genes16101184)
Supplement: Supplementary file 1 [file genes-16-01184-s001.zip › genes-3834835-supplementary.pdf]

Supplementary Table S1 PARMS Primer sequence

SNP Marker	Physical Position	Variation Locus	Primer sequence (5′-3′)
M1	24009393	A/T	Rt:GAAGGTGACCAAGTTCATGCTCGGTGCTGAAAGGGAGTGT<br>Ra:GAAGGTCGGAGTCAACGGATTCGGTGCTGAAAGGGAGTGA<br>F:TGTAGCTTCGAACTGCCAACA
M2	25993581	G/C	Rc:GAAGGTGACCAAGTTCATGCTGGAAAGGCGAAAACTTTACTACAC<br>Rg:GAAGGTCGGAGTCAACGGATTGGAAAGGCGAAAACTTTACTACAG<br>F:GCTCCTTTGTTAATTTAGGGCTAAT
M3	26987124	G/A	Rc:GAAGGTGACCAAGTTCATGCTGATACACTAGCCTCACCCTGTCC<br>Rt:GAAGGTCGGAGTCAACGGATTAGATACACTAGCCTCACCCTGTCT<br>F:GGATGAGCTCCTTAAAGTCTGTAAC
M4	28034065	C/T	Fc:GAAGGTGACCAAGTTCATGCTAAAGTCCCCAACACAATCCTC<br>Ft:GAAGGTCGGAGTCAACGGATTAAAGTCCCCAACACAATCCTT<br>R:AATCCTAAACCAAGAACAGCGA
M5	28991377	C/G	Fc:GAAGGTGACCAAGTTCATGCTCATCTTACAATACCTATACAAGTTTCC<br>C<br>Fg:GAAGGTCGGAGTCAACGGATTCATCTTACAATACCTATACAAGTTTC<br>CG<br>R:CTTGAAATCGCTTTGTCCACATA
M6	29990089	T/C	Ra:GAAGGTGACCAAGTTCATGCTTGCGTGACAATGAAGAGATCAGTA<br>Rg:GAAGGTCGGAGTCAACGGATTGCGTGACAATGAAGAGATCAGTG<br>F:ATGCTGGTTAGCGTTCCAGAC
M7	30100772	C/A	Rg:GAAGGTGACCAAGTTCATGCTGGTAAAATCCATGGTATATCACTCG<br>Rt:GAAGGTCGGAGTCAACGGATTGGGTAAAATCCATGGTATATCACTCT<br>F:AAATAATTTCAGAATATGACCGGAG
M8	30402945	T/A	Ra:GAAGGTGACCAAGTTCATGCTCACACACTTGAATTTTAAACTTCACA<br>Rt:GAAGGTCGGAGTCAACGGATTTCACACACTTGAATTTTAAACTTCACT<br>Rc:AGGGATAGTTACTGATCGAGTACCC
M9	30600586	A/G	Fa:GAAGGTGACCAAGTTCATGCTCCCGAATTAGAAAGTCGTGAGAA<br>Fg:GAAGGTCGGAGTCAACGGATTCCCGAATTAGAAAGTCGTGAGAG<br>R:CGCATTTTCGTGTCAATTAAGTG
M10	30820924	T/C	Fa:GAAGGTGACCAAGTTCATGCTCCCGAATTAGAAAGTCGTGAGAA<br>Fg:GAAGGTCGGAGTCAACGGATTCCCGAATTAGAAAGTCGTGAGAG<br>R:CGCATTTTCGTGTCAATTAAGTG
M11	31150266	A/T	Rt:<br>GAAGGTCGGAGTCAACGGATTGCAAATAAAGTCTTGATCTCTTTCTCA<br>Rc:GAAGGTGACCAAGTTCATGCTTTCTAAATCCTAAATTCTAAACCCAAT<br>F:CTCAATTATAAACTCTAAACCTAAATTCAG
M12	31180314	A/T	Fc:GAAGGTGACCAAGTTCATGCTGATCCGTCCTCAGCACGC<br>Ra: GAAGGTGACCAAGTTCATGCTGCAAATAAAGTCTTGATCTCTTTCTCT<br>F: CTTATCAAATCATATCATAGCCAATGT

SNP Marker	Physical Position	Variation Locus	Primer sequence(5′-3′)
M13	31209409	A/G	Rt:GAAGGTGACCAAGTTCATGCTCTCATACCTCACTGAACGATTCCT<br>Rc:GAAGGTCGGAGTCAACGGATTCATACCTCACTGAACGATTCCC<br>F:CAATAACACTTACCCAAGCCCT
M14	31304070	G/A	Rg:GAAGGTGACCAAGTTCATGCTGAGACGAAATGCTGGAAGAAGG<br>Ra:GAAGGTCGGAGTCAACGGATTGAGACGAAATGCTGGAAGAAGA<br>F:TAATTTGATACTACGGTGCAACCC
M15	31409624	T/C	Ra:GAAGGTGACCAAGTTCATGCTTGTCTTCTCATAGCAGCGACAA<br>Rg:GAAGGTCGGAGTCAACGGATTTGTCTTCTCATAGCAGCGACAG<br>F:AAGCTAATAAGCAAATGGATGAGAC
M16	31521617	T/A	Ft:GAAGGTGACCAAGTTCATGCTTCGTCCACTAGACATTCTTTTCTCT<br>Fa:GAAGGTCGGAGTCAACGGATTTCGTCCACTAGACATTCTTTTCTCA<br>R:ACTATCAACAGAGTGAATGGGAGAA
M17	31632032	T/C	Ft:GAAGGTGACCAAGTTCATGCTTCATACCAGGTAAATGATAATAGTGTT<br>TT<br>Fc:GAAGGTCGGAGTCAACGGATTTCATACCAGGTAAATGATAATAGTGT<br>TTC<br>R:GGAAATTTTGCAGCAAGCG
M18	31655211	C/T	Fc:GAAGGTGACCAAGTTCATGCTTGAAGAGCATGCAGAGTAGGTAGTC<br>Ft:GAAGGTCGGAGTCAACGGATTTGAAGAGCATGCAGAGTAGGTAGTT<br>R:ATCTGGAAAGGTTCAAGGATCG
M19	31686432	G/A	Fc:GAAGGTGACCAAGTTCATGCTGCAAAGACAAACCAAAGCTCG<br>Fa:GAAGGTCGGAGTCAACGGATTGCAAAGACAAACCAAAGCTCA<br>R:TCTCCGCAAGAGTGGTACACAA

Supplementary Table S2 One hundred forty five recombinants and their genotypes detected in F_2 population

Mark name	M1	M2	M3	M4	M5	M6	M19	Phenotype
Physical location	24009393	25993581	26987124	28034065	28991377	29990089	316816432	-
P1	A	A	A	A	A	A	A	purple
P2	B	B	B	B	B	B	B	green
F1	H	H	H	H	H	H	H	purple
11-C11	H	H	H	H	H	H	B	green
11-D05	H	H	H	H	H	H	B	green
1-A06	H	H	H	H	H	H	B	green
5-A06	H	H	H	H	H	H	B	green
5-F10	H	H	H	H	H	H	B	green
6-D01	H	H	H	H	H	H	B	green
6-D06	H	H	H	H	H	H	B	green
11-H06	H	H	H	H	H	H	B	green
11-A01	H	H	B	B	B	B	B	green
11-A04	H	B	B	B	B	B	B	green
11-A05	H	B	B	B	B	B	B	green
11-A06	H	B	B	B	B	B	B	green
11-A07	H	H	B	B	B	B	B	green
11-A08	H	B	B	B	B	B	B	green
11-A09	H	B	B	B	B	B	B	green
11-A10	H	B	B	B	B	B	B	green
11-A11	H	B	B	B	B	B	B	green
11-A12	H	H	H	B	B	B	B	green
11-B01	H	B	B	B	B	B	B	green
11-B02	H	H	B	B	B	B	B	green
11-B03	H	H	H	B	B	B	B	green
11-B04	H	B	B	B	B	B	B	green
11-B06	H	H	H	B	B	B	B	green
11-B08	H	H	B	B	B	B	B	green
11-B11	H	H	B	B	B	B	B	green
11-B12	H	H	H	H	H	B	B	green
11-C01	H	B	B	B	B	B	B	green
11-C02	H	B	B	B	B	B	B	green
11-C03	H	B	B	B	B	B	B	green
11-C04	H	H	H	B	B	B	B	green
11-C05	H	H	B	B	B	B	B	green
11-C06	H	H	B	B	B	B	B	green
11-C07	H	B	B	B	B	B	B	green

Mark name	M1	M2	M3	M4	M5	M6	M19	Phenotype
Physical location	24009393	25993581	26987124	28034065	28991377	29990089	316816432	-
11-C08	H	B	B	B	B	B	B	green
11-C09	H	B	B	B	B	B	B	green
11-C10	H	H	B	B	B	B	B	green
11-C12	H	H	H	B	B	B	B	green
11-D01	H	H	B	B	B	B	B	green
11-D02	H	H	B	B	B	B	B	green
11-D03	H	H	H	B	B	B	B	green
11-D04	H	H	B	B	B	B	B	green
11-D06	H	B	B	B	B	B	B	green
11-D07	H	H	B	B	B	B	B	green
11-D08	H	H	H	H	H	B	B	green
11-D09	H	B	B	B	B	B	B	green
11-D10	H	B	B	B	B	B	B	green
11-D11	H	B	B	B	B	B	B	green
11-D12	H	B	B	B	B	B	B	green
11-E01	H	B	B	B	B	B	B	green
11-E02	H	H	B	B	B	B	B	green
11-E03	H	H	H	H	B	B	B	green
11-E04	H	B	B	B	B	B	B	green
11-E05	H	H	H	B	B	B	B	green
11-E06	H	H	B	B	B	B	B	green
11-E07	H	B	B	B	B	B	B	green
11-E08	H	H	H	H	B	B	B	green
11-E09	H	B	B	B	B	B	B	green
11-E10	A	B	B	B	B	B	B	green
11-E11	H	H	B	B	B	B	B	green
11-F04	H	H	B	B	B	B	B	green
11-F05	H	H	B	B	B	B	B	green
11-F08	H	H	B	B	B	B	B	green
11-F09	H	B	B	B	B	B	B	green
11-F11	H	H	B	B	B	B	B	green
11-F12	H	H	H	B	B	B	B	green
11-G01	H	H	H	B	B	B	B	green
11-G03	H	B	B	B	B	B	B	green
11-G05	H	H	H	H	B	B	B	green
11-G07	H	B	B	B	B	B	B	green
11-G08	H	H	B	B	B	B	B	green
11-G09	H	H	H	B	B	B	B	green
11-G10	H	H	H	B	B	B	B	green

Mark name	M1	M2	M3	M4	M5	M6	M19	Phenotype
Physical location	24009393	25993581	26987124	28034065	28991377	29990089	316816432	-
11-G11	H	B	B	B	B	B	B	green
11-H02	H	H	H	B	B	B	B	green
11-H03	H	B	B	B	B	B	B	green
11-H04	H	B	B	B	B	B	B	green
11-H05	H	H	H	H	B	B	B	green
11-H07	H	H	H	H	B	B	B	green
11-H08	H	B	B	B	B	B	B	green
12-A02	H	B	B	B	B	B	B	green
12-A03	H	B	B	B	B	B	B	green
12-A04	H	H	B	B	B	B	B	green
12-A05	H	B	B	B	B	B	B	green
12-A06	H	B	B	B	B	B	B	green
12-A07	H	B	B	B	B	B	B	green
12-A08	H	B	B	B	B	B	B	green
12-A09	H	B	B	B	B	B	B	green
12-A10	H	B	B	B	B	B	B	green
12-A11	H	B	B	B	B	B	B	green
12-A12	H	B	B	B	B	B	B	green
12-B01	H	H	H	B	B	B	B	green
12-B02	A	B	B	B	B	B	B	green
12-B04	H	B	B	B	B	B	B	green
12-B06	H	H	B	B	B	B	B	green
12-B07	H	B	B	B	B	B	B	green
12-B08	H	B	B	B	B	B	B	green
12-B09	H	B	B	B	B	B	B	green
12-B10	H	H	B	B	B	B	B	green
12-B11	H	H	H	B	B	B	B	green
12-C01	H	H	H	H	B	B	B	green
12-C02	H	B	B	B	B	B	B	green
12-C03	H	B	B	B	B	B	B	green
12-C04	H	H	B	B	B	B	B	green
12-C05	H	H	H	H	B	B	B	green
12-C06	H	H	B	B	B	B	B	green
12-C07	H	H	B	B	B	B	B	green
12-C09	H	H	B	B	B	B	B	green
12-C10	H	B	B	B	B	B	B	green
12-C11	H	H	B	B	B	B	B	green
12-C12	H	H	H	B	B	B	B	green
12-D01	H	H	H	H	B	B	B	green
12-D02	H	B	B	B	B	B	B	green

Mark name	M1	M2	M3	M4	M5	M6	M19	Phenotype
Physical location	24009393	25993581	26987124	28034065	28991377	29990089	316816432	-
12-D03	B	B	B	B	B	H	B	green
12-D04	H	B	B	B	B	B	B	green
12-D05	H	B	B	B	B	B	B	green
12-D06	H	H	B	B	B	B	B	green
12-D07	H	B	B	B	B	B	B	green
12-D08	H	B	B	B	B	B	B	green
12-D09	H	H	H	B	B	B	B	green
12-D10	H	B	B	B	B	B	B	green
12-D11	H	B	B	B	B	B	B	green
12-D12	H	H	B	B	B	B	B	green
12-E01	H	H	B	B	B	B	B	green
12-E02	H	B	B	B	B	B	B	green
12-E03	H	H	H	B	B	B	B	green
12-E05	H	B	B	B	B	B	B	green
12-E06	H	B	B	B	B	B	B	green
12-E07	H	H	H	H	B	B	B	green
12-E08	H	H	B	B	B	B	B	green
12-E10	H	B	B	B	B	B	B	purple
12-E12	H	B	B	B	B	B	B	purple
12-F01	B	B	B	B	B	H	H	purple
12-F04	B	B	B	B	H	H	H	purple
12-F05	B	B	H	H	H	H	H	purple
12-F08	B	H	H	H	H	H	H	purple
12-F09	H	H	B	B	B	B	H	purple
12-F11	B	H	H	H	H	H	H	purple
12-F12	B	H	H	H	H	H	H	purple
12-G02	B	B	B	B	H	H	H	purple
12-G04	B	B	H	H	H	H	H	purple
12-G05	B	B	H	H	H	H	H	purple
12-G07	B	H	H	H	H	H	H	purple
12-G08	B	B	B	H	H	H	H	purple
12-G12	B	H	H	H	H	H	H	purple
12-H01	B	H	H	H	H	H	H	purple

Note: A: Homozygous dominant; B: Homozygous recessive; H: Heterozygous
